# Characterization of Flavan-3-ols and Expression of *MYB* and Late Pathway Genes Involved in Proanthocyanidin Biosynthesis in Foliage of *Vitis bellula*

**DOI:** 10.3390/metabo3010185

**Published:** 2013-03-19

**Authors:** Yue Zhu, Qing-Zhong Peng, Ci Du, Ke-Gang Li, De-Yu Xie

**Affiliations:** 1 Hunan Provincial Key Laboratory of Plant Resources Conservation and Utilization, College of Biology and Environmental Sciences, Jishou University, No.120 Ren Min Nan Lu, Jishou City, Hunan Province, 416000, China; E-Mail: lkg@jsu.edu.cn (K.-G. L.); 2 Department of Plant Biology, North Carolina State University, 100 Derieux Place, Raleigh, NC 27695, USA

**Keywords:** *Vitis bellula*, proanthocyanidins, anthocyanidin reductase, leucoanthocyanidin reductase, flavan-3-ols

## Abstract

Proanthocyanidins (PAs) are fundamental nutritional metabolites in different types of grape products consumed by human beings. Although the biosynthesis of PAs in berry of *Vitis vinifera* has gained intensive investigations, the understanding of PAs in other *Vitis* species is limited. In this study, we report PA formation and characterization of gene expression involved in PA biosynthesis in leaves of *V. bellula*, a wild edible grape species native to south and south-west China. Leaves are collected at five developmental stages defined by sizes ranging from 0.5 to 5 cm in length. Analyses of thin layer chromatography (TLC) and high performance liquid chromatography-photodiode array detector (HPLC-PAD) show the formation of (+)-catechin, (−)-epicatechin, (+)-gallocatechin and (−)-epigallocatechin during the entire development of leaves. Analyses of butanol-HCl boiling cleavage coupled with spectrometry measurement at 550 nm show a temporal trend of extractable PA levels, which is characterized by an increase from 0.5 cm to 1.5 cm long leaves followed by a decrease in late stages. TLC and HPLC-PAD analyses identify cyanidin, delphinidin and pelargonidin produced from the cleavage of PAs in the butanol-HCl boiling, showing that the foliage PAs of *V. bellula* include three different types of extension units. Four cDNAs, which encode *VbANR*, *VbDFR*, *VbLAR1* and *VbLAR2*, respectively, are cloned from young leaves. The expression patterns of *VbANR* and *VbLAR2* but not *VbLAR1* and *VbDFR* follow a similar trend as the accumulation patterns of PAs. Two cDNAs encoding VbMYBPA1 and VbMYB5a, the homologs of which have been demonstrated to regulate the expression of both *ANR* and *LAR* in *V. vinifera*, are also cloned and their expression profiles are similar to those of *VbANR* and *VbLAR2*. In contrast, the expression profiles of *MYBA1* and *2* homologs involved in anthocyanin biosynthesis are different from those of *VbANR* and *VbLAR2*. Our data show that both ANR and LAR branches are involved in PA biosynthesis in leaves of *V. bellula*.

## 1. Introduction

Proanthocyanidins (PAs), also known as condensed tannins, are oligomeric or polymeric flavan-3-ols. In general, PAs play important protective roles for plants against damages caused by pathogens, herbivores and UV irradiation [1]. In addition, the presence of PAs in forage crops, such as alfalfa, can protect ruminant animals from pasture bloating disease. More importantly, the routine consumption of PAs from food and drink products, such as green tea, grape and cranberry, can prevent people from cancer, cardiovascular and aging diseases [1,2].

*V. vinifera* and *V. rotundifolia*, which are two species widely cultivated for wine and fruit products, have gained numerous investigations to understand PAs and other phenolic molecule properties that contribute fundamental nutritional benefits to human health [3,4,5,6,7,8,9]. Wines have been demonstrated to have multiple benefits. Particularly, the “French Paradox” story [10] has led to intensive studies in order to understand the mechanism of cardiovascular benefits of red wine. A great number of research efforts over the past many years have demonstrated that oligomeric PAs and their monomers such as (+)-catechin and (−)-epicatechin are one main group of flavonoid metabolites in red and white wines and form main beneficial substance [11,12,13,14,15]. The uptake of PAs and flavan-3-ols from wine or grape juice products can generally prevent human being from cardiovascular diseases, such as heart diseases, atherosclerosis, ischemic reperfusion injury, and lowering low density lipoprotein (LDL) levels [9,13,16,17]. When rats were fed with PAs, their hearts were shown a high resistance to myocardial ischemia reperfusion injury [17]. Grape seed-derived PAs fed to rabbits were identified in plasma, in which their antioxidative activity significantly decreased a severe atherosclerosis in the aorta through a mechanism of reducing oxidation of LDL [12]. In addition, the addition of PA-rich extract to human plasma has been shown to inhibit the oxidation of cholesteryl linoleate in LDL [12].

**Figure 1:**
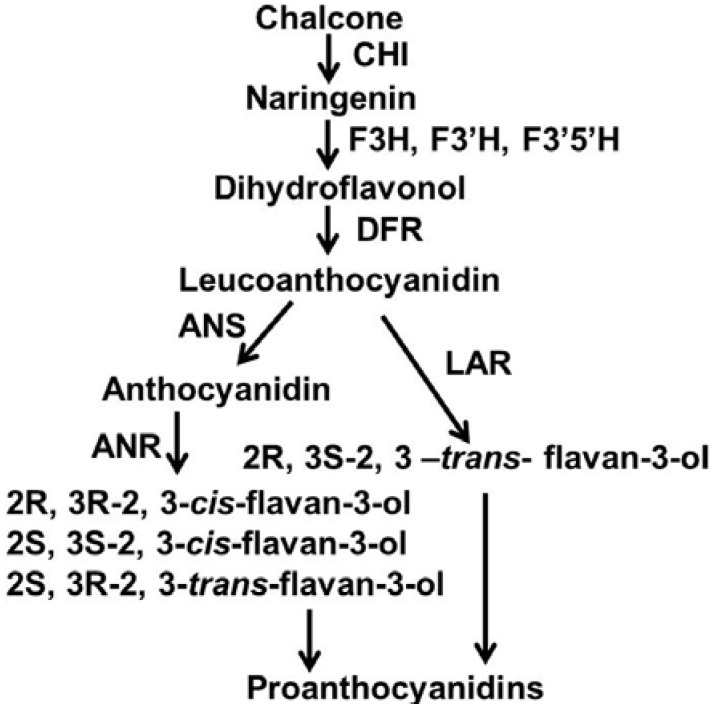
The biosynthetic pathway of grape proanthocyanidins starting with chalcone. CHI: chalcone isomerase; F3H, flavanone-3-hydroxylase; F3'H, flavonoid 3'-hydroxylase; F3'5'H, flavonoid 3',5'-hydroxylase; DFR, dihydroflavonol reductase; LAR, leucoanthocyanidin reductase; ANS, anthocyanidin synthase; ANR, anthocyanidin reductase.

The PA biosynthesis in berries of *V. vinifera* is tightly associated with molecular properties of flavan-3-ols that commonly exist in wine and grape juice products. Over the past decade, multiple intensive studies in functional genomics and biochemistry have greatly enhanced the elucidation of the biosynthetic pathway of PAs in grape berries. Particularly, the late pathway has been characterized by two independent branches starting with leucoanthocyanidins leading to different isomers of flavan-3-ols, e.g. (+)-catechin and (−)-epicatechin. The biogenesis of (+)-catechin is catalyzed by a leucoanthocyanidin reductase (LAR), the enzyme of which uses 2R, 3S-2, 3-*trans*-3, 4-*cis*-leucocyanidin as substrate [18]. This branch is termed the LAR pathway of PAs [19]. The biogenesis of (-)-epicatechin and (−)-catechin is catalyzed by an anthocyanidin reductase (ANR), the enzyme of which uses non-chiral cyanidin as substrate [20]. This branch is termed the ANR pathway of PAs [19]. In the genome of *V. vinifera*, genes encoding LAR and ANR have been cloned. Biochemical studies and crystallization of enzymes have demonstrated that the LAR and ANR pathways [19] (Figure 1) co-exist in grapes leading to the formation of flavan-3-ols and PAs [21,22]. More importantly, several intensive studies on both crystal structure and enzymatic mechanisms have demonstrated that VvLAR specifically converts 2R, 3S-2, 3-*trans*-3, 4-*cis*-leucoanthocyanidins to the 2R, 3S-*trans*-flavan-3-ol isomers, e.g. (+)-catechin. In contrast, VvANR has been demonstrated to have a dual function characterized by a reduction and an epimerase activity. VvANR converts anthocyanidins to 2R, 3R-*cis*-flavan-3-ols and then catalyzes epimerization to form 2S, 3R-*trans*- and 2S, 3S-*cis*-flavan-3-ols, such as (-)-epicatechin, (−)-catechin and (+)-epicatechin (Figure 1) [23]. These discoveries elucidate the enzymatic origin of 4 types of absolute configurations of flavan-3-ols in berries of grapes.

In comparison with *V. vinifera*, studies on PA biosynthesis in other species of *Vitis* are lacking. In addition, the understanding on PAs and other polyphenolics in foliage of grape plants are particularly limited. To date, only a few experiments have been carried out to characterize PA biosynthesis in leaves of *V. vinifera* [22,24,25]. Although results from these studies are limited, those experimental data enhance a better understanding of PAs in *V. vinifera* plants. We believe that continuous studies on leaves likely enhance the possibility of revealing new structures, such as (−)-epiafzelechin and (+)-afzelechin, which has never been reported in berries of *V. vinifera*. In addition, any new information of PAs in foliage of different grape species can promote improvement of nutritional values of berries and wine products.

**Figure 2:**
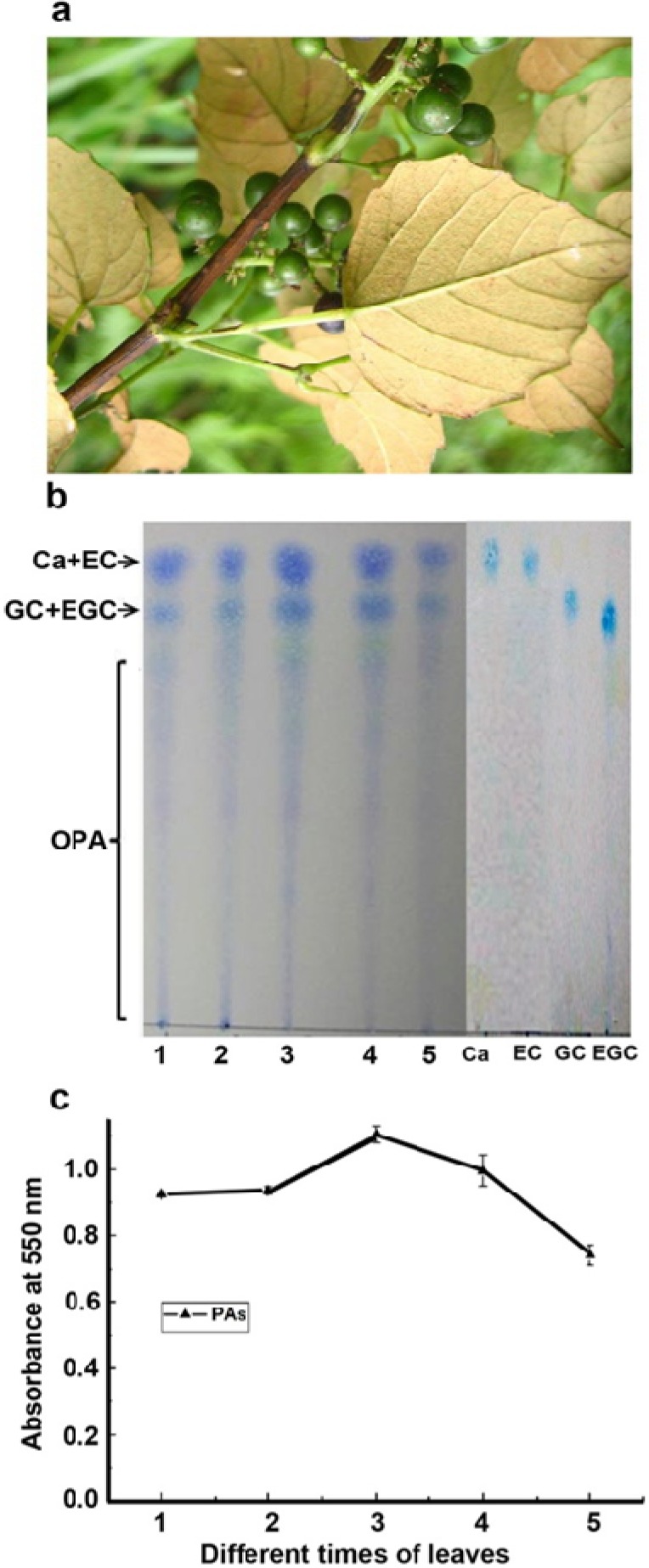
An image of leaves and green berries of *Vitis bellula* and analysis of PAs in leaves at different development stages. (**a**). morphologies of leaves and unripen green berries; (**b**). images of PA profiles visualized by 0.1% DMACA on a TLC plate, Ca+EC: (+)-catechin and (−)-epicatechin, GC+EGC: (-)-gallocatechin and (−)-epigallocatechin, OPA: oligomeric PAs; (**c**). comparison of PA levels at different times of leaf development from young (numerated as 1) through fully expanded time (numerated as 5), 1: less than 0.5 cm, 2: 0.5–1.0 cm, 3: 1.0–1.5 cm, 4: 1.5–2.0 cm and 5: longer than 2.0 cm to fully expanded (4–5 cm in length). Crude extracts of PAs were boiled in butanol: HCl 1 hr to produce anthocyanidins, the absorbance values of which were measured at 550 nm.

*V. bellula* natively grows in south and southwest China. Its ripe berries are consumed by local Chinese people because of their high nutritional values. To better understand PA structures and biosynthesis in *V. bellula*, we are interested in investigating both leaves and berries. In present study, we report characterization of main flavan-3-ols and molecular properties of PA extension units as wells as gene expression involved in the late pathways in leaves of *V. bellula* (Figure 2a).

## 2. Results and Discussion

### 2.1. Characterization of Proanthocyanidin Formation at Different Stages of Leaf Development

TLC assay is a simple and effective method to visualize flavan-3-ols and extractable PAs (E-PAs) [26]. In this study, TLC was performed to examine profiles of PAs in leaf extracts in methanol. As reported previously [19,26], DMACA was used to visualize monomers, oligomeric PAs and polymers. Blue coloration resulted from the reaction of DMACA and flavan-3-ols and PAs showed profiles and relative abundance in 5 µL methanol of leaf extracts. The profile image shown by visualization determined the presence of flavan-3-ols with the same R_f_ values as standards of (±)-catechin, (−)-epicatechin, (−)-gallocatechin and (−)-epigallocatechin (Figure 2b). Oligomeric PAs with different R_f_ values were also extracted from leaf tissues (Figure 2b). These data indicated that flavan-3-ols and PAs are synthesized during different times of leaf development.

Butanol-HCl boiling coupled with UV-spectrometry analysis is an effective method to estimate levels of PAs in plant tissues [19,26]. After the boiling of PAs in butanol-HCl reagent, estimation of OD values at 550 nm showed a dynamic trend of E-PAs in leaf tissues from 0.5 cm through 5 cm in length. The levels of E-PAs were similar in 0.5–1 cm long leaves, followed by a significant increase in 1.0–2.0 cm long leaves, then significantly decreased in 2.0–3.0 cm and 3.0–5.0 cm long leaves (Figure 2c). This trend supported the intensity changes of blue coloration images of DMACA staining showed in TLC plates (Figure 2b). The decrease trend of PA levels in stages 4 and 5 likely resulted from dilution as leaf expansion or the reduction of biosynthetic pathway activity.

### 2.2. Characterization of Flavan-3-ols and Extension Units of Proanthocyanidins

**Figure 3:**
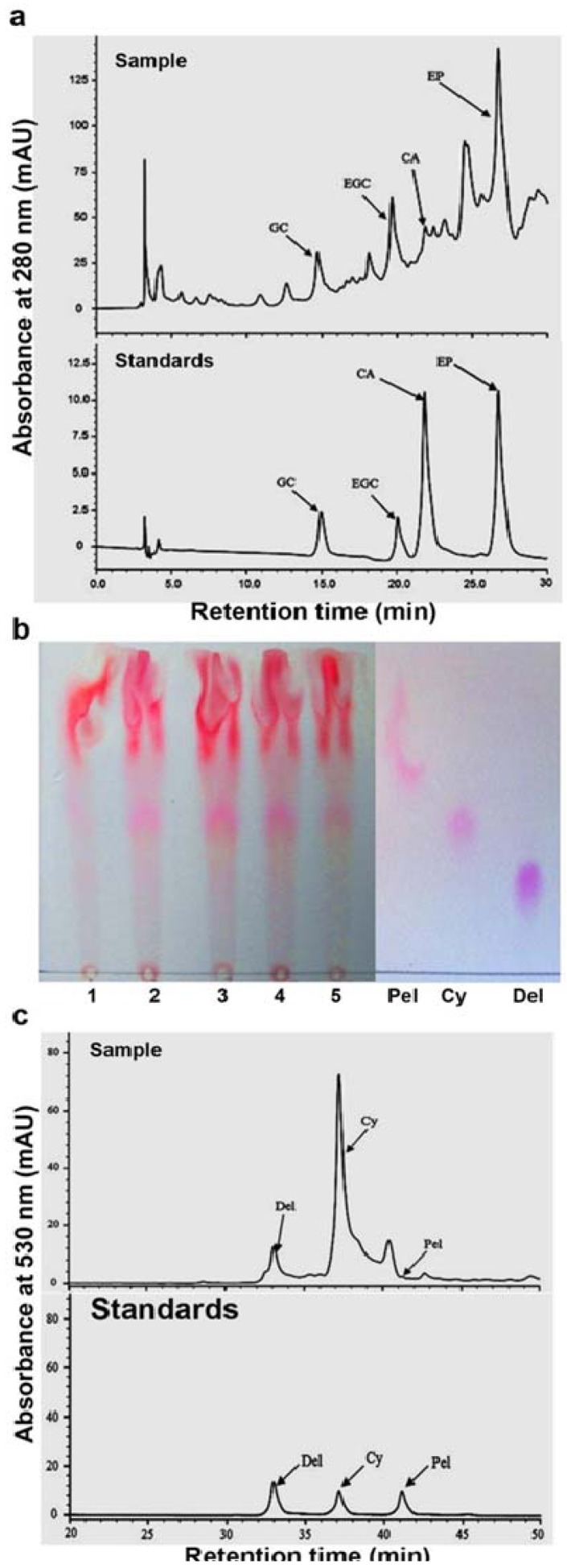
HPLC profiling of flavan-3-ols and TLC analysis of proanthocyanidins in leaves of *Vitis bellula.* (**a**). A HPLC profile shows (±)-catechin (CA), (-)-epicatechin (EP), (−)-gallocatechin (GC) and (−)-epigallocatechin (EGC) in crude extracts of leaves (stage 3); (**b**). A TLC image shows anthocyanidin profiles released from the butanol: HCl boiling of crude extracts of proanthocyanidins from leaves (stage 3), authentic standards including: cyanidin (Cy), pelargonidin (Pel) and delphinidin (Del); (**c**). A HPLC profile shows anthocyanidins released from the butanol: HCl boiling of crude extracts of proanthocyanidins from leaves (stage 3), authentic standards including: cyanidin (Cy), pelargonidin (Pel) and delphinidin (Del).

HPLC-PAD analysis coupled with the use of authentic standards has been shown an effective approach to characterize flavan-3-ols [19,26]. In this study, leaf extracts in methanol were analyzed by HPLC-PAD. Chromatography of separated metabolites was recorded at 280 nm to characterize flavan-3-ols. Four peaks were identified with the same retention times as authentic standards, (+)-catechin, (−)-epicatechin, (−)-gallocatechin and (−)-epigallocatechin, respectively (Figure 3a). These four peaks were also showed the same UV spectra as authentic standards, respectively. These data show that 2, 3-*trans* and *cis*-flavan-3-ols are synthesized in leaves.

HPLC-PAD and TLC analyses have been used to characterize products derived from the butanol-HCl boiling of PAs [19,26]. Red pigments produced in the butanol-HCl boiling of PAs were separated on cellulose-based TLC plates. Components that were at the positions with the same R_f_ values as authentic standards of cyanidin, delphinidin and pelargonidin were determined in leaf extracts from different developmental stages (Figure 3b). This result demonstrated that these three red pigment metabolites were produced from the butanol-HCl boiling of PAs. In addition, other red pigment components with higher R_f_ values than pelargonidin R_f_ value were obviously detected in leaf extracts (Figure 3b). To further demonstrate the properties of red pigments, metabolites separated by HPLC was recorded at 550 nm to compare their retention time and UV-spectra with authentic standards. The resulting data showed that three peaks were eluted at the same retention times (Figure 3c) and exhibited the same spectra (data not shown) as cyanidin, pelargonidin and delphinidin, respectively. Therefore, cyanidin, delphinidin and pelargonidin were released from the butanol-HCl boiling cleavage of PAs, demonstrating that the extension units of PAs are composed of 2-(3',4'-dihydroxyphenyl)-3,4- dihydro-2H-chromene-3,5,7-triol, e.g. (+)-catechin or (-)-epicatechin; 2-(4-hydroxyphenyl)-3,4- dihydro- 2H-chromene -3,5,7-triol, e.g. (+)-afzelechin or (-)-epiafzelechin; and 2-(3',4',5'-hydroxyphenyl) -3,4-dihydro-2H-chromene-3,5,7-triol, e.g. (+)-gallocatechin or (-)-epigallocatechin.

It was interesting that, in addition to cyanidin, delphinidin and pelargonidin, there were additional red pigment components detected from the butanol-HCl boiling of PAs by both TLC and HPLC (Figure 3b,c). Images of TLC showed that main red pigments were characterized by higher R_f_ values in comparison with cyanidin, delphinidin and pelargonidin. Meanwhile, HPLC showed two main additional peaks between cyanidin and pelargonidin. Although the properties of these additional pigments remains further identified, these results reveal that additional structures likely exists in PAs of leaves.

### 2.3. Expression Patterns of ANR, LAR and DFR Homologs During Leaf Development

**Figure 4:**
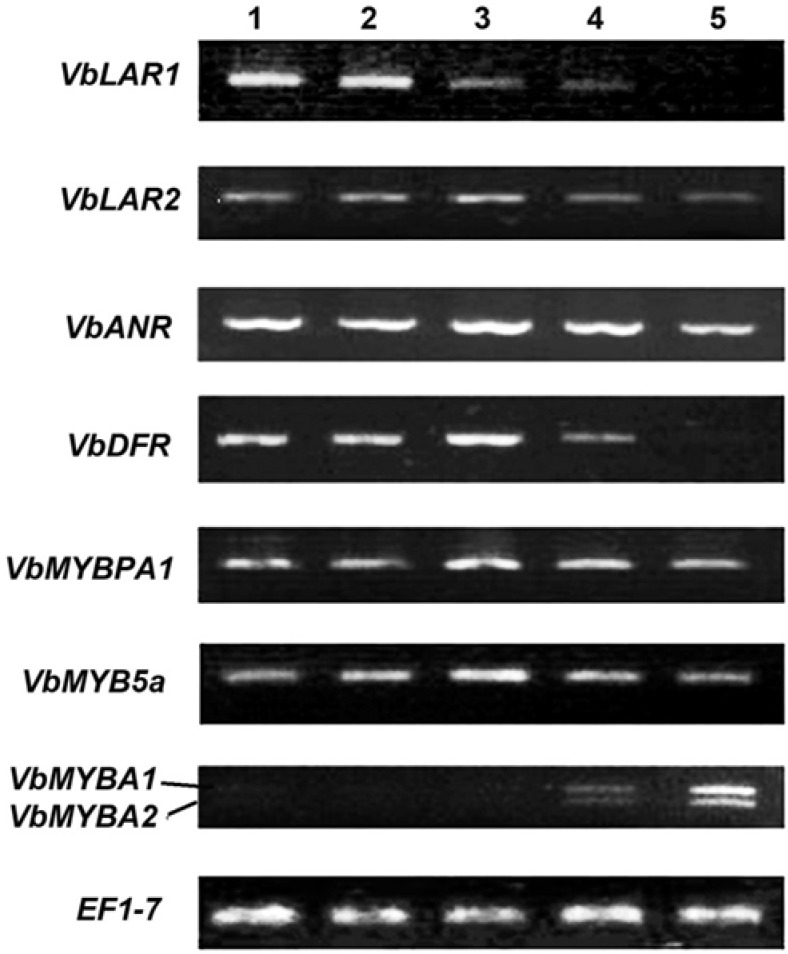
Images of semi quantitative RT-PCR. RT-PCR results show expression patterns of three late pathway genes and three MYB genes at five points in the leaf development of *V. bellula*. *ANR*: anthocyanidin reductase, *LAR*: leucoanthocyanidin reductase, *DFR*: dihydroflavonol reductase, *MYBA1*, *A2* and *5a*: MYB transcription factors, *EF1-γ*: elongation factor gamma as reference gene.

ANR from *V. vinifera* has been functionally and in crystal characterized to convert anthocyanidins to three different isomers of flavan-3-ols featured by configurations of 2R, 3R-2,3-*cis*, 2S, 3S-*cis* and 2S, 3R-*trans*-flavan-3-ols, e.g. (−)-epicatechin, (+)-epicatechin and (−)-catechin (Figure 1) [22]. Based on the sequence of *V. vinifera ANR* (*Vv ANR*), one pair of degenerated primers (Table 1) was created to amplify *ANR* homologs from leaves of *V. bellula*. The open reading frame (ORF) were cloned and termed as *VbANR* in this study. Its sequence (gi|381392348|gb|JQ308620.1) was shown to possess very high similarity to *VvANR* [19]. Semi-quantitative PCR analysis showed that its expression level was higher at stage #3 than at any of the other four stages in the leaf development (Figure 4), which supported the observation of the peak value of E-PAs at the stage 3 and consistent synthesis of flavan-3-ol isomers, e.g. (−)-epicatechin and (−)-epigallocatechin extracted in methanol and (Figure 2b and Figure 3a). 

**Figure 5:**
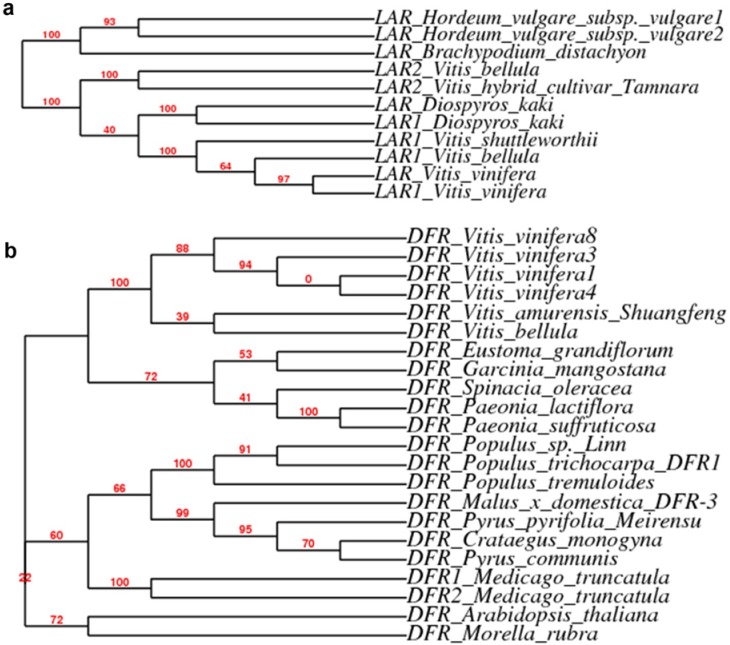
Cladogram style phylogeny trees of *DFR* and *LAR* from certain species. (**a**). a phylogeny tree developed from 11 cDNA homolog sequences of *LAR*. (**b**). a phylogeny tree developed from selected 22 cDNA homolog sequences of *DFR*.

Two *LAR* members, *VvLAR1* and *2*, have been identified in the genome of *V. vinifera* [22]. These two isomers have been shown to convert 2R, 3S-2, 3-*trans*-3, 4-*cis*-leucocyanidin to (+)-catechin. Based on sequences of *VvLAR 1* and *2*, degenerated premier pairs (Table 1) were designed to amplify homologs from leaves of *V. bellula*. Consequently, two ORF cDNA fragments were amplified and sequence analysis showed that these two homologs were highly similar to *VvLAR1* and *2*, respectively. As result, we termed these two *LAR* homologs as *VbLAR1* and *2* and deposited these two sequences to the gene bank curated at NCBI (*VbLAR1*: gi|381392360|gb|JQ308626.1|; *VbLAR2*: gi|381392362|gb|JQ308627.1|). A phylogeny analysis with 11 *LAR* homolog sequences revealed that *VbLAR1* and *VvLAR1* were in the same clade, while *VbLAR2* and *VvLAR2* were in the same clade (Figure 5a). Semi-quantitative RT-PCR analysis revealed differentiated expression patterns of these two genes. The expression of *VbLAR1* occurred in the early times but was hardly detected in the late times of leaf development. The expression level of *VbLAR2* was similar at stages #1 to #2 and then increased at stage #3 followed by a decrease at stages #4 and 5 of leaf development (Figure 4).

An ORF cDNA fragment of *DFR* homolog was amplified by PCR and its sequence (>gi|381392350|gb|JQ308621.1|) was deposited to the gene bank curated at NCBI. We termed this cDNA as *VbDFR*. Semi-quantitative RT-PCR analysis showed that its expression levels were similar at stages #1 and 2, and then increased at stage #3 followed by a decrease at stage #4, but hardly detected at stage #5 during leaf development (Figure 4). A phylogeny analysis with 22 *DFR* homologs revealed that *VbDFR* and other homologs from *Vitis* species were in the same clade (Figure 5b).

### 2.4. Expression Patterns of VbMYBPA1, VbMYB5a, VbMYBA1 and VbMYBA2 During Leaf Development

*VvMYBPA1* cloned from berries of *V. vinifera* encodes a R2R3-MYB member, a homolog of TT2 regulating PA biosynthesis in seeds of *A. thaliana* [25]. A cDNA homolog of *VvMYBPA1* was amplified from leaves of *V. bellula*, which is termed *VbMYBPA1*. Its ORF sequence (gi|381392354|gb|JQ308623.1) has been deposited in the gene bank curated at NCBI. Semi-quantitative RT-PCR analyses showed that the expression level of *VbMYBPA1* was higher at stage 3 than those at any of the other four stages tested during leaf development (Figure 4), which was consistent to the trend of the PA level (Figure 2c). Given that VvMYBPA1 has been demonstrated to specifically regulate PA biosynthesis by controlling promoter activities of *VvLAR* and *VvANR* [21,25,27], we hypothesize that *VbMYBPA1* most likely is involved in the regulation of PA biosynthesis in leaves of *V. bellula*. 

A cDNA homolog of *VvMYB5a* was amplified from leaves of *V. bellula*, which is termed as *VbMYB5a*. We have deposited its OFR sequence (gi|381392352|gb|JQ308622.1|) to the gene bank curated at NCBI. Semi-quantitative RT-PCR analyses showed that the expression level of *VbMYB5a* was higher at stage 3 than those at any of the other four stages tested (Figure 4), the trend of which was corresponding to that of PA levels (Figure 2c). *VvMYB5a* has been cloned and demonstrated to encode another R2R3-MYB member regulating the general phenylpropanoid pathway. Its overexpression leads to production of anthocyanins and flavonols with a cost of lignin alternation in transgenic tobacco plants [28]. The expression patterns of *VvMYB5a* have been demonstrated to be consistent to the expression of *VvLAR* and *VvANR* in the beginning of the development of berries [21]. As a result, we hypothesize that *VbMYB5a* is likely involved in PA and other flavonoid biosynthesis in leaves of *V. bellula*.

Two cDNA homologs of the grape *VvMYBA* family were amplified from leaves of *V. bellula* using one pair of primers (Table 1 and Figure 4). The full sequence of ORF for one cDNA was obtained and deposited to the public gene bank (gi|381392354|gb|JQ308623.1|) curated at NCBI. Sequence blast analysis showed that this ORF sequence was highly similar to the ORF of *VvMYBA1*, which is one main member of the small *VvMYBA* family regulating anthocyanin biosynthesis associating with red and white berry features of different *V. vinifera* varieties [29]. Here, this cDNA homolog was termed *VbMYBA1*. In addition, the second PCR fragment amplified was smaller than *VbMYBA1* (Figure 4)*.* Sequence analysis showed that it had approximately 90% of the identity compared to *VvMYBA2*, which is another member of the grape *VvMYBA* family, and thus was termed *VbMYBA2* in this study. Semi-quantitative RT-PCR analysis showed that these two DNAs shared a very similar expression pattern, which was featured by from undetectable levels at stage #1 to a relatively high expression level at stage #5 tested in leaf development (Figure 4). Multiple studies have shown that VvMYBA1 likely is a master regulator associating with red pigmentation in berries of grapes and VvMYBA2 is also involved in certain specific accumulation of anthocyanin pigmentation [29,30,31,32,33]. We hypothesize that *VbMYBA1* and *2* are also involved in anthocyanin biosynthesis and other flavonoids biosynthesis in leaves of *V. bellula*.

### 2.5. Discussion

*Vitis* has approximately 60 species [34]. In addition to *V. vinifera*, 15 other species are cultivated for berry products and wine production, such as *V. rotundifolia* that is another economically important crop. To date, only berries of *V. vinifera* have gained most intensive investigations to understand biosynthesis of PAs. This is because the biosynthetic properties and structures of PAs in berries of this species are fundamentally associated with nutritional values of grape berries and quality of wine products [15,35,36,37]. Numerous investigations have showed that the structures of PAs in wine products made from berries of *V. vinifera* are mainly characterized by procyanidins consisting of either (+)-catechin or (−)-epicatechin or both [6,12,38,39,40,41]. In contrast, little has been reported about the presence of (+)-afzelechin (derived from leucopelargonidin) or (−)-epiafzelechin (derived from pelargonidin) units in grape seed PAs. Several experiments have demonstrated that grape skins synthesize pelargonidin-glycosides [42,43,44,45,46]. These discoveries imply that (−)-epiafzelechin units likely exist in grape PAs. In addition, compared to berries, leaves have gained very limited studies to understand PA biosynthesis. Meanwhile, whether leaves can produce propelargonidin or miscellaneous oligomeric or polymeric PAs consisting of (−)-epiafzelechin and (−)-epicatechin remains unclear. We believe that studies on berries and leaves of different species of *Vitis* can enhance a better understanding of properties of PA biosynthesis and structures in this genus. In present study, the butanol-HCl boiling of crude PAs in leaf extract of *V. bellula* released pelargonidin (Figure 3b,c). These results imply the presence of (−)-epiafzelechin or (+)-afzelechin units in foliage PAs of *V. bellula* because these units after butanol-HCl boiling are converted to pelargonidin [1,19]. As more experiments for elucidation of PA structures using LC-MS and NMR analyses will be carried out, (−)-epiafzelechin or (+)-afzelechin units in PAs can be characterized in the future. In addition to pelargonidin, cyanidin and delphinidin were produced from the butanol-HCl boiling of crude PAs (Figure 3b,c), showing that epicatechin or catechin and epigallocatechin or gallocatechin are two other types of extension units. These results indicate that the foliage PAs contain extension units featured with one, two and three groups of –OH in the B-ring of the flavan-3-ols, potential examples of which are epiafzelechin, (−)-epicatechin and (−)-epigallocatechin. Furthermore, both HPLC and TLC analyses show additional pigment molecules (Figure 3b,c). Although the structures of these pigment molecules remain to be elucidated, our data indicate the structural diversity of PAs in leaves of *V. bellula*.

In this study, we focus on gene expression analysis of late pathway genes (*VbDFR*, *VbANR*, *VbLAR1 and VbLAR2*) of PA biosynthesis. ORFs of cDNAs for these four genes were sequenced for phylogeny analysis. Our previous phylogenetic analysis showed that all available nucleotide sequences of *ANR* cDNAs in the *Vitis* genus were clustered in the same clade [19]. Here, a phylogenetic analysis also showed that *VvLAR1* and *VbLAR1* were in the same clade, while *VbLAR2* and *VvLAR2* were in the same clade (Figure 5a). In addition, *VbDFR* was in the same clade as *VvDFR* and *VaDFR* cloned from *V. vinifera* and *V. amurensis*, respectively (Figure 5b). These analyses show that the nucleotide sequences of *DFR*, *ANR* and *LAR* ORF cDNAs are conserved in the *Vitis* genus. Semi-quantitative analyses have been performed to understand their expression patterns during leaf development examined from young to fully expanded stages (Figure 4). Based on intensity of amplified cDNA fragment indicated by signal of EB-DNA binding (Figure 4), the expression patterns of *VbANR*, *VbDFR* and *VbLAR2* were corresponding to the trend of levels of PAs extracted in methanol (Figure 2c). Our observation on *VbANR* and *VbLAR2* expression patterns is different from the report about the *VvANR* and *VvLAR2* expression patterns in leaf development of *V. vinifera* [22]. In our experiments, the expression pattern of *VbANR* and *VbLAR2* was characterized by a similar level at the stages 1 and 2, followed by a level increase at the stage 3 and then a level decrease at stages 4 and 5 (Figure 4). The expression pattern of *VvANR* in leaves of *V. vinifera* was featured by a trend consisting of a level decrease from stage 1 to stage 2 and then a continuous level increase from stage 2 to stage 5 [22]. The expression pattern of *VvLAR2* was characterized by a trend of a continuous decrease from stage 1 to stage 4 and then an increase at stage 5 [22]. Unlike *VbANR* and *VbLAR2*, the expression of *VbLAR1* followed a unique pattern in leaf development, which was featured by relatively high levels in the early two stages followed by a decrease to an undetectable level at stage 5 (Figure 4). This expression pattern is also different from that of *VvLAR1* that was showed a very low expression level in leaves of *V. vinifera* [22]. We propose two possibilities associating with these different expression patterns between two species. On the one hand, these distinct expression patterns in two species are likely associated with different patterns of biosynthesis of PAs and sampling times. On the other hand, these differences most likely result from two genotypes and other environmental conditions in their growth areas. More importantly, our data show that the ANR and LAR pathways co-exist in leaves of *V. bellula*. In summary, these observations support the general hypothesis that the co-existence of the ANR and LAR pathways of PA biosynthesis is common biochemical phenomenon in the vascular plants [19]. To date, in addition to *V. vinifera* and *V. bellula*, many vascular plants, such as tea (*Camellia sinensis*) [47], apple [24], *Gingko biloba* [48] and other species [19], have been demonstrated to include these two branches toward the formation of PAs.

The expression patterns of four MYB transcription factor homologs (*VbMYBPA1*, *VbMYB5a*, *VbMYBA1* and *VbMYBA2*) involved in biosynthesis of grape PAs and anthocyanins were analyzed by semi-quantitative RT-PCR (Figure 4). VbMYBPA1 is a homolog of VvMYBPA1, which has been demonstrated to regulate the expression of both *VvANR* and *VvLAR1*, but not *VvLAR2* during berry development of *V. vinifera* [25,49]. In our study, we also observed that the expression pattern of *VbMYBPA1* was similar to those of *VvANR* and *VvLAR2*, but not *VvLAR1* (Figure 4). *VvMYB5a* encodes a R2R3-MYB member that has been demonstrated to regulate several branches of the phenylpropanoid pathway leading to PAs, anthocyanins and flavonols during berry development of *V. vinifera* [21,28,50]. The expression pattern of *VvMYB5a* has been shown to associate that of *VvLAR1* [21]. In our study, a *VbMYB5a* with a high sequence similarly to *VvMYB5a* was cloned from leaves of *V. bellula*. Its expression profile was very similar to *VbANR*, *VbLAR2* and *VbMYBPA1* (Figure 4), indicating that *VbMYB5a* most likely is associated with the regulation of *VbANR* and *VbLAR2*. Two R2R3-MYB homologs associated with anthocyanin biosynthesis, VbMYBA1 and VbMYBA2, were identified from berry of *V. vinifera* [29,30,31,32,33,51,52]. These two transcription factors regulate the biosynthesis of anthocyanins in the skin of berries. RT-PCR analysis revealed the expression of two homologs, *VbMYBA1* and *VbMYBA2*, in late two stages but not in the early three stages of leaf development examined (Figure 4). Compared to the expression patterns of *VbLAR* and *VbANR*, this result does not indicate an involvement of these two transcription factors in the PA biosynthesis. In addition, their expression profiles were not consistent with that of *VvDFR*. Based on these observations, we suggest that *VbMYBA1* and *2* in leaf of *V. bellula* are involved in regulation of other branches of the phenylpropanoid pathway.

## 3. Experimental Section

### 3.1. Plant Materials

*V. bellula* natively grows in the mountainous area of Jishou (28°19'0"N, 109°43'0"E) in the district of west Hunan province, China. Leaves at 5 different developmental stages were collected from 5–6 years old vines in late spring, 2010 and 2011, respectively. The developmental stages were defined on the basis of the leaf lengths after initiation from auxiliary buds, including approximately 0.5 cm (stage #1), 0.5–1.0 cm (stage #2), 1.0–2.0 cm (Stage #3), 2.0–3.0 cm (stage #4) and 3.0–5.0 cm (stage #5) in length, respectively. Detached leaves were immediately frozen in liquid nitrogen and stored −70–80 °C until experimental uses described below.

### 3.2. Extraction of Proanthocyanidins And Flavan-3-ols and TLC Assay

Frozen leaf samples were ground into fine powder in liquid nitrogen. Two hundred milligrams of powder were weighed into a 1.5 mL Eppendorf tube and then suspended in 1 mL of acetone (100%, Sigma, USA). The tube was vortexed 30 second, followed by 20 min sonication. The tube was centrifuged 5 min at 6,000 rpm. The supernatant phase of acetone was pipetted into a 15 mL polyethylene tube. This extraction step was repeated twice and the three-time acetone extractions were pooled together in the same tube to gain a final volume of approximately 3 mL. This acetone extraction was dried in a speed-vacuum centrifuge. One and half mL of extraction was pipetted into a new 1.5 mL Eppendorf tube, which was centrifuged at 3000 rpm under a reduced vacuum till the removal of acetone. Then the left 1.5 mL acetone extraction was dried off in the same tube with the same procedure. The remained pellet at the bottom of tube was suspended in 500 μL deionized water (from MilliQ) followed by addition of 200 μL chloroform (100%). The mixture was vortexed 2 min and then centrifuged 5 min at 10000 rpm to gain the upper water and bottom chloroform phases, the latter of which included fatty acids, lipids and chlorophyll and was pipetted into a waste bottle. This step was repeated once. The left water phase and interphase in the tube was added 500 μL ethyl acetate (EA). The tube was vortexed 2 min followed by 5 min centrifugation at 10,000 rpm to gain the upper EA phase and the bottom water phase. The EA phase including PAs, flavan-3-ols and other flavonoids was pipetted into a new 1.5 mL Eppendorf tube. This EA extraction step was repeated twice to gain 1.4 mL of EA extraction. Then, EA was removed with the same speed-vacuum method. The remained residue in the bottom of the tube was dissolved in 500 μL methanol. After 5 min centrifugation at 10,000 rpm, the methanol extraction was pipetted into a new 1.5 mL tube for high performance liquid chromatography (HPLC) and TLC analyses of PA and flavonols described below. All of steps were performed in room temperature.

A thin layer chromatography (TLC) assay was performed to profile PA and flavan-3-ols. Five µL of leaf extract in methanol was loaded onto a glass-backed silica TLC plate (0.2 mm thickness). Ten µL of (−)-gallocatechin, (−)-epigallocatechin, (+)-catechin, and (−)-epicatechin (purchased from sigma) dissolved in methanol (0.1 µg/µL) were loaded onto the plate as authentic standards of reference. The protocol for separation of metabolites and visualization of PAs and flavan-3-ols with DMACA was as described previously [19].

### 3.3. Butanol: HCl Boiling of Proanthocyanidins, Absorbance Measurement and TLC Assay

The method of butanol: HCl boiling of PAs was as described previously [26]. In brief, 50 μL of methanol extract was added into 950 μL reagent composed of butanol: HCl (95: 5, V/V) and mixed thoroughly, followed by boiling 1 hr in a water bath. After cooling to approximately room temperature, the boiled mixture was used to measure optical density values (OD) at 550 nm on a spectrophotometer (model UV-255, Shimadzu, Japan). After measurement, 1 mL of the boiled mixture was dried off using a rotary speed-vacuum evaporator at room temperature. The remained residue was dissolved in 50 μL methanol containing 0.1% HCl followed by a centrifugation of 5 min at 10,000 rpm. The supernatant was pipetted into a new 1.5 mL tube for TLC and HPLC analysis of anthocyanidins described below.

TLC assay was performed to examine anthocyanidins released from the butanol: HCl boiling of PAs. The TLC plate used was cellulose F-200µM chromatography layer (purchased from Liangchengguiyuan, China). Authentic standards (5 μL) of cyanidin chloride, delphinidin chloride and pelargonidin chloride (purchased from Sigma) were loaded onto the plate as reference. The protocol of separation was as described previously [26].

### 3.4. HPLC Analysis of Flavan-3-ols and Anthocyanidins

High performance of liquid chromatography (HPLC) was performed on a Shimadzu LC-20AT (Shimadzu, Japan) instrumentation equipped with an SPD-M20A photodiode array detector. Metabolites were separated in a reversed-phase Diamonsil C18 column (150 × 4.6 mm, 5 µm). To characterize aglycones of flavan-3-ols in leaf extract of methanol, metabolites were separated with a gradient elution program formed by solvent A (0.1% phosphoric acid in double deionized water) and solvent (100% acetonitrile, HPLC grade). The gradient program was composed of series of ratios of solvent A to solvent B: 95:5 (0–5 min), 95:5 to 90:10 (5–10 min), 90:10 to 83:17 (10–25 min), 83:17 to 77:23 (25–30 min), 77:23 to 50:50 (30–65 min), 50:50 to 0:100 (65–69 min), 0:100 (69–79 min) and 0:100 to 95:5 (79–80 min), then followed by a 10 min post washing. The chromatography was recorded at 280 nm. The injection volume was set at 10 µL of leaf extract of methanol. The flow rate was set up at 1 mL per min. (−)-Gallocatechin, (−)-epigallocatechin, (+)-catechin, and (-)-epicatechin dissolved in methanol (0.1 µg/µL) were injected as authentic standards of reference. The volume injected for each standard was set at 5 µL. To analyze anthocyanidins released from the butanol-HCl boiling of PAs, another elution gradient program was developed to consist of a series of ratios of solvent A to B: 90:10 (0–5 min), 90:10 to 85:15 (5–10 min), 85:15 to 72:28 (10–15 min), 72:28 to 50:50 (15–45 min), 50:50 to 0:100 (45–50 min), 0:100 (50–60 min), 0:100 (69–79 min) and 0:100 to 90:10 (60–70 min), then followed by 10 min post washing. Anthocyanidins were recorded at 550 nm as described previously [26]. The injection volume of samples was set at 10 µL. The flow rate was set up at 1 mL per min. Cyanidin chloride, delphinidin chloride and pelargonidin chloride (purchased from Sigma) prepared in 0.1% HCl methanol (0.1 µg/µL) were used as authentic standard reference, respectively. The volume injected for each standard was set at 5 µL.

### 3.5. Total RNA Isolation and Semi-Quantitative RT-PCR Analysis

One gram of frozen leaf tissues at different development stages was used to isolate total RNA using Plant Total RNA Isolation Kits (purchased from Sangon, Shanghai, China). The isolation procedures were followed the manufacturer's protocol. Each RNA sample was treated with DNAase (provided in Kits to remove potential genomic DNA). One microgram of DNA-free RNA was used as template for reverse transcription reaction to synthesize the first strand of cDNA via using BluePrint^®^ 1st strand cDNA Synthesis Kit (including MMLV RTase) (Cat#: 6115A Takara, Japan) following the manufacturer's protocol.

**Table 1:** Primer pairs are designed to amplify cDNAs of eight genes involved in proanthocyanidin biosynthesis and *Ef1–7* in leaves of *V. bellula*.

cDNAs	Sequence 5'-3'	Thermal Cycle
*VbLAR1*	F: ATGACTGTTTCTCCGGTTCCTTCG	94 °C 5 min, 30cycles of 94 °C 30 sec, 58 °C 30 sec and 72 °C 45 sec, 72 °C 10 min
	R: TCAAGCGCAGGTTGCAGTGAC	
*VbLAR2*	F: ATGACTGTTTTGTCTGTGAGTACTC	94 °C 5 min, 30cycles of 94 °C30 sec, 55 °C 30 sec and 72 °C 45 sec, 72 °C 10 min
	R: TCAGGCGCAGGTAGCAGTGATG	
*VbANR*	F: ATGGCCACCCAGCACCCCAT	94 °C 5 min, 30cycles of 94 °C30 sec, 55°C 30 sec and 72 °C 45 sec, 72 °C 10 min
	R: TCAATTCTGCAATAGCCCCTTGGC	
*VbDFR*	F: ATGGGTTCACAAAGTGAAACCGTG	94 °C 5 min, 30cycles of 94 °C30 sec, 50 °C 30 sec and 72 °C 45 sec, 72 °C 10 min
	R: CTAGGTCTTGCCATCTACAGG	
*VbMYBPA1*	F: ATGGGCAGAGCACCTTGTTG	94 °C 5 min, 30cycles of 94 °C30 sec, 50 °C 30 sec and 72 °C 45 sec, 72 °C 10 min
	R: TTAAATGAGTAGTGATTCGGCG	
*VbMYB5a*	F: ATGAGGAATGCATCCTCAGCATCAG	94 °C 5 min, 30cycles of 94 °C30 sec, 55 °C 30 sec and 2 °C 45 sec, 72 °C 10 min
	R: TCAGAACCGCTTATCAGGTTGATCG	
*VbMYBA1 and 2*	F: ATGGAGAGCTTAGGAGTTAGAAAG	94 °C 5 min, 30cycles of 94 °C30 sec, 47 °C 30 sec and 72 °C 45 sec, 72 °C 10 min
	R:TCAGATCAAGTGATTTACTTGTG	
*EF1-γ*	F: GCGGGCAAGAGATACCTCAA	94 °C 5 min, 30cycles of 94 °C30 sec, 50 °C 30 sec and 72 °C 30 sec, 72 °C 10 min
	R:TCAATCTGTCTAGGAAAGGAAG	

Semi-quantitative PCR was performed to amplify cDNAs of three late pathway genes including dihydroflavonol reductase, anthocyanidin reductase, and leucoanthocyanidin reductase genes. In addition, cDNA fragments of four MYB homologs were amplified. Primers (Table 1) of these seven cDNAs were designed based on their homologs reported in the genome of *V. vinifera*. In addition, a pair of primers was designed to amplify cDNA fragment of *VvEF1-r*, a housing keeping gene, to evaluate quantity of the first cDNA used in each PCR. All reagents for PCR were included in the same kit of RT. The reaction volume was set at 25 μL. Each step of PCR was followed the manufacturer's protocol. The thermal reaction programs for amplification of eight cDNAs and *VvEF-r* are included in Table 1. The products of PCR were visualized using ethidium bromide after electrophoresis on 1% agarose gels.

### 3.6. Sequence Analysis

PCR products were cloned to a T-easy vector (CloneTech) for sequencing, which was completed at BGI (Beishan Industrial Zone, Yantian District, Shenzhen, 518083, China). Open reading frames of eight cDNAs (*VbDFR, VbLAR1, VbLAR2, VbANR, VbMYBPA1, VbMYB5a, VbMYBA1* and *VbMYBA2*) were used for sequence blastn analysis and then were deposited in the gene bank at NCBI. Sequences of *VbDFR* and its homologs as well as sequences of *VbLAR1* and *VbLAR2* and their homologs were upload to Phylogeny.fr (Available online: http://www.phylogeny.fr/version2_cgi/index.cgi) [53] to construct phylogenetic trees. 

### 3.7. Statistical Analysis

Student *T-*test (*p* value < 0.01) was performed to evaluate significant difference of PA levels among leaf tissues estimated by UV spectrometry.

## 4. Conclusion

To date, berries of *V. vinifera* have gained most intensive investigations to understand structures and biosynthesis of PAs and flavan-3-ols. In contrast, leaf tissues of *V. vinifera* and other species have gained very limited studies to understand PA biosynthesis. To better understand PA structures and biosynthesis in *Vitis* species, studies on different tissues of other species are necessary. This type of investigation can also improve PA compositions in grape berries and wine products. In this study, we particularly report that leaves of *V. bellula* synthesize flavan-3-ols such as (+)-catechin, (−)-epicatechin, (−)-gallocatechin and (−)-epigallocatechin. This result demonstrates that leaves express two late branches, the ANR and LAR pathways to flavan-3-ols and PAs. In addition, two regulation genes, *VbMYBPA* and *VbMYB5a*, are expressed in leaves regulating the ANR and LAR pathways. Furthermore, the butanol-HCl boiling cleavage of PAs produces pelargonidin, cyanidin and delphinidin. This result demonstrates that PAs are composed of at least three types of flavan-3-ols characterized by one, two or three –OH groups in the B-ring of extension units.
